# Molecular Characterizations of Subcellular Localization Signals in the Nucleocapsid Protein of Porcine Epidemic Diarrhea Virus

**DOI:** 10.3390/v6031253

**Published:** 2014-03-13

**Authors:** Da Shi, Maojie Lv, Jianfei Chen, Hongyan Shi, Sha Zhang, Xin Zhang, Li Feng

**Affiliations:** Division of Swine Infectious Diseases, State Key Laboratory of Veterinary Biotechnology, Harbin Veterinary Research Institute, Chinese Academy of Agricultural Sciences, Harbin 150001, China; E-Mails: dashi198566@163.com (D.S.); lvmaojie650@sina.com (M.L.); chenjianfei@126.com (J.C.); shy2005y@163.com (H.S.); zs8838566@126.com (S.Z.); zhangxin2410@163.com (X.Z.)

**Keywords:** CHROMOSOME region maintenance 1, nucleocapsid protein, porcine epidemic diarrhea virus, subcellular localization signals

## Abstract

The nucleolus is a dynamic subnuclear structure, which is crucial to the normal operation of the eukaryotic cell. The porcine epidemic diarrhea virus (PEDV), coronavirus nucleocapsid (N) protein, plays important roles in the process of virus replication and cellular infection. Virus infection and transfection showed that N protein was predominately localized in the cytoplasm, but also found in the nucleolus in Vero E6 cells. Furthermore, by utilizing fusion proteins with green fluorescent protein (GFP), deletion mutations or site-directed mutagenesis of PEDV N protein, coupled with live cell imaging and confocal microscopy, it was revealed that, a region spanning amino acids (aa), 71–90 in region 1 of the N protein was sufficient for nucleolar localization and R87 and R89 were critical for its function. We also identified two nuclear export signals (NES, aa221–236, and 325–364), however, only the nuclear export signal (aa325–364) was found to be functional in the context of the full-length N protein. Finally, the activity of this nuclear export signal (NES) was inhibited by the antibiotic Lepomycin B, suggesting that N is exported by a chromosome region maintenance 1-related export pathway.

## 1. Introduction

Porcine epidemic diarrhea (PED) was first recognized as a devastating enteric disease in feeder and fattening pig, resembling transmissible gastroenteritis (TGE) in pigs in the United Kingdom, in 1971. It is a member of *Coronavirinae*, which are single-stranded, positive-sense RNA viruses with the largest genome that are known to infect humans, other mammals, and birds, usually causing subclinical or respiratory and gastrointestinal diseases. The porcine epidemic diarrhea virus (PEDV) subgenomic mRNAs, which are transcribed from the genome, produce viral proteins, such as the spike (S, 180–220 kDa), envelope (E, ~8.8 kDa), membrane (M, 27–32 kDa), nucleoprotein (N, 55–58 kDa), and several other proteins of unknown function [1,2,3]. Among the proteins, N, as the RNA-binding protein, play an important role in both virus RNA synthesis and modulating host cell processes, and phosphorylation may regulate these processes by exposing various functional motifs [4,5]. Several other functions have been postulated for the coronavirus N protein throughout the virus life cycle, including encapsidation, packaging, correct folding of the RNA molecule, the deregulation of the host cell cycle [6,7,8], inhibition of interferon production [9,10], up-regulation of COX2 production [11,12], up-regulation of AP1 activity [13], induction of apoptosis [14,15,16], association with host cell proteins [17], and RNA chaperone activity [18]. Therefore, it is clear that N is a multifunctional protein involved in biological processes related to the survival of PEDV.

The nucleolus was one of the first subcellular structures to be identified by early users of the light microscope, appearing as a highly refractive black dot(s) in the nucleus of the cell [19]. The nucleolus is a highly specialized structure that participates in regulation of several host cell processes, including ribosome subunit biogenesis, RNA processing, control of cell growth and response [20]. Interestingly, a cytoplasmic-nucleolar distribution pattern has been reported for the N proteins of several coronaviruses, including representative members of *Alphacoronavirus* (transmissible gastroenteritis virus, TGEV), *Betacoronavirus* (mouse hepatitis virus, MHV; and severe acute respiratory syndrome coronavirus, SARS-CoV), and *Gammacoronavirus* (infectious bronchitis virus, IBV) [2,21,22]. Further study indicated that N protein co-localize with major nucleolar proteins, including nucleolin, fibrillarin, and nucleophosmin [23,24,25].

How viral and cellular proteins traffic to the nucleolus and what determines their sub-nucleolar localization is not clearly understood, but proteins that localize to the cytoplasm and nucleus or nucleolus contain multiple signals that determine their subcellular localization [26], such as nucleolar localization signal (NoLS). Active nuclear import of proteins is mediated by nuclear localization signals (NLSs), which are then recognized by proteins of the importin super-family (importin α and β) that mediate the transport across the nuclear envelope using Ran-GTP [27]. Similar to nuclear import, export of a protein from nucleus depends on the presence of a specific nuclear export signal (NES) [28]. The chromosome region maintenance 1 (CRM1; also known as exportin 1 or Xpo1) has been identified as an export receptor that interacts with the predominant NES, the so-called leucine-rich NES, which is found in a large variety of nucleocytoplasmic shuttling proteins [29,30]. In fact, some of these NESs are not necessarily leucine rich but rather characterized by several hydrophobic residues. The pharmacological compound leptomycin B (LMB) directly interacts with CRM1 and blocks NES-mediated protein export [31]. Therefore, the proteins can shuttle between the nucleus and the cytoplasm with their subcellular localization signals.

It was reported previously that N protein nucleolar localization is a common feature in coronaviruses, however, there are different results regarding N subcellular localization in a strain of SARS-CoV [4]. Within the *Alphacoronavirus* coronaviruses, the precise NoLS and NES of PEDV N and its traffic mechanism are still elusive. Therefore, we have attempted to characterize these signals, and the molecular mechanism responsible for its subcellular localization. In this study, we examined the intracellular localization of the PEDV N protein in PEDV-infected and transfected cell lines using mouse polyclonal antisera and confocal microscopy. By generating a series of deletion and mutagenesis constructions, we found that amino acids 71-90 in region 1 were sufficient for nucleolar retention and we also identified two NESs (aa221–236 and 325–364), but only the NES (aa325–364) was found to be functional in the context of the full-length N protein. The nucleocytoplasmic shuttling of N and the nuclear export of GFP-NES could be blocked by LMB, an inhibitor of the CRM1, which is the receptor for exportin-1-dependent nuclear export. 

## 2. Results and Discussion

### 2.1. Polyclonal Antibody React Specifically with the N Protein of PEDV

**Figure 1:**
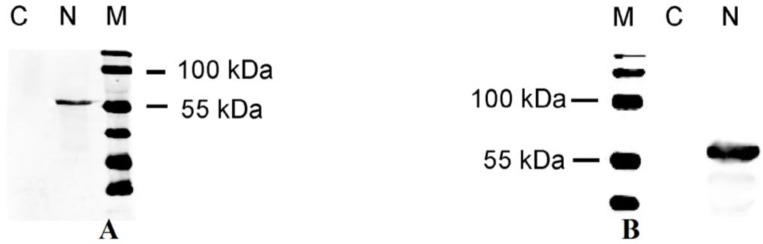
(**A**) Characterization of the mouse anti-N polyclonal antibody. Lane 1, negative control; Lane 2, N protein from PEDV CV777; M, protein marker; (**B**) Analysis of N protein in transiently transfected Vero E6 cells. M, protein marker; Lane 1, pcDNA3.1 (+) control; Lane 2, N protein.

A polyclonal antibody specifically against the N protein was produced to determine its intracellular localization. We generated anti-N mouse antisera using an E coli-produced fusion protein as the antigen. The antigenicity of the recombinant N protein was confirmed by immunoreactivity with PEDV pigs sera using ELISA assay, which showed high sensitivity and specificity (data not show). To examine the reactivity and specificity of the mouse antiserum, blot results demonstrated that mouse antisera notably reacted with N protein from CV777 strain PEDV, and the cell lysate from Vero E6 cells transfected with pcDNA3.1-N showed a band with the same molecular mass to N protein, whereas no band was detected from samples of cells uninfected PEDV and transfected with an empty vector alone (Figure 1A,B). 

### 2.2. N Protein Can Localize in Nucleolus in PEDV-Infected and Transfected Cell Lines

**Figure 2:**
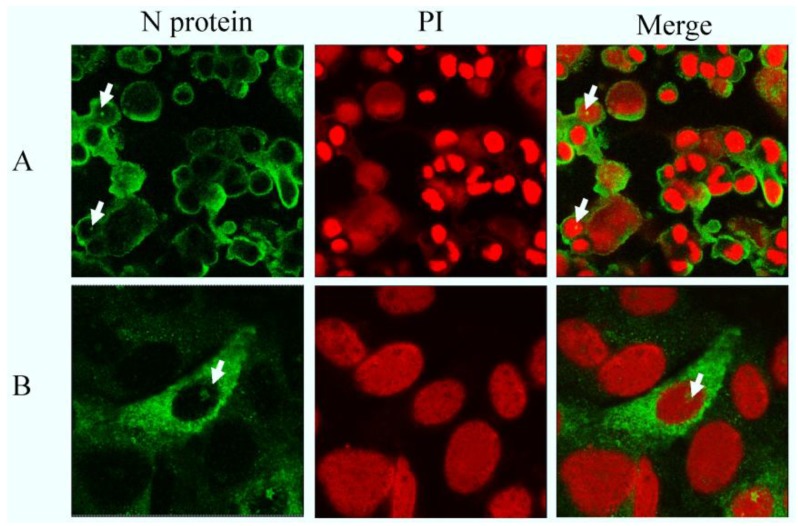
(**A**) Detection of PEDV N protein by indirect immunofluorescence in cells infected with PEDV CV777; (**B**) Detection of PEDV N protein by indirect immunofluorescence in cells transfected with pcDNA3.1 (+)-N. After 24 h, infected or transfected, cells were fixed and analyzed by indirect immunofluorescence using mouse anti-N polyclonal antibody (Green) and stained with PI (red) to visualize the nuclear DNA. Differentially fluorescing images were gathered separately using confocal microscopy. Images were obtained with a 63× oil objective.

Our results showed that N protein was localized predominantly in the cytoplasm in PEDV CV777-infected cells, while in a few cells fluorescence was also observed in the nucleus (or nucleolus) (Figure 2A). No significant fluorescence was observed in uninfected cells (data not shown). A similar observation was also found in Vero E6 cells transfected with plasmid expressing full length N protein (Figure 2B). The N protein was observed to localize mainly in the cytoplasm with some protein in the nucleus (or nucleolus). The results suggested that the N protein localized to a subnuclear structure and may contain functional signals. To identify predicted nuclear (or nucleolar) localization signals, and whether they participate in this process or not, we conducted further experiments.

### 2.3. Bioinformatics Analysis of Subcellular Localization Signals and Delineation of a NoLS in Region 1 of PEDV N Protein

**Figure 3:**
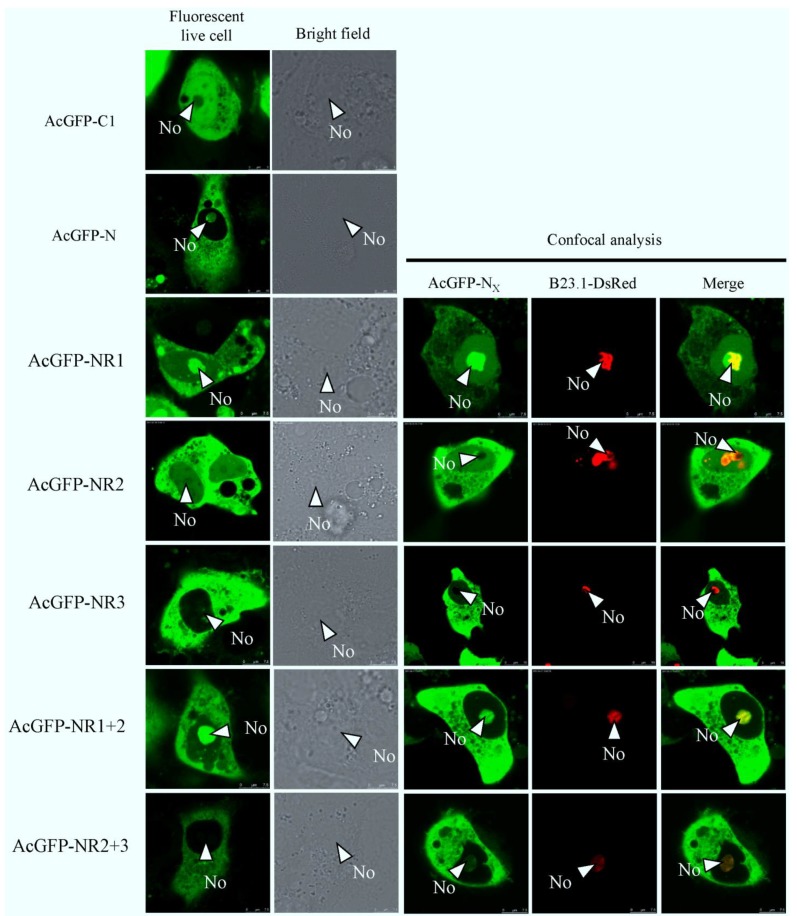
Live cell and confocal microscopy of the subcellular localization of fluorescent fusion proteins: AcGFP, AcGFP-N, AcGFP-NR1, AcGFP-NR2, AcGFP-NR3, AcGFP-NR1+2, and AcGFP-NR2+3 proteins. Vero cells were visualized 24 h post-transfection in culture conditions. Confocal analysis of the subcellular localization of AcGFP, AcGFP-N, AcGFP-NR1, AcGFP-NR2, AcGFP-NR3, AcGFP-NR1+2, and AcGFP-NR2+3 proteins in cells co-expressing B23.1-DsRed, at 24 h post-transfection. The PEDV fusion peptides are colored green and the B23.1 fusion protein colored red. Merged images are also presented. The nucleolus (No) is arrowed where appropriate.

To identify whether there were subcellular localization signals in PEDV N protein, we first conducted a bioinformatics analysis on the protein using existing motif prediction algorithms. Predict NLS [32] and PSORT II [33] were used to identify potential NLSs, and the NES predictor (Net NES) [29] was used to identify potential NESs. Predict NLS found no NLSs, whereas PSORT II indicated that PEDV N protein contained a pat7 motif (261PKKNKSR267). Net NES found no NES. In other studies, coronaviruses, such as TGEV, MHV, IBV, and SARS-CoV showed a common characteristic, that of NLS-rich in *C*-terminal. So through amino acid sequence comparison among groups we also found a basic amino acid-rich short peptide (383RKKEKKNKRE393) in C-terminal. We presumed it might play a role in N protein localization as a new localization signal, and named it patx. Although N protein contains putative nuclear (or nucleolar) localization signals (N (or No) LS), it is not known whether they are functional or not. To investigate whether these and other unknown signals operated to determine the subcellular trafficking of N protein, we utilized AcGFP as a fusion marker to observe the localization characteristics of N and truncated N protein in live cells. The B23.1-DsRed fusion protein was used to tag the nucleolus, so we could analyze nucleolar localization properties and colocalization in cotransfected cells by live cell imaging (direct fluorescence) or confocal microscopy. At 24 h post-cotransfection, live cell imaging indicated that as previously shown, AcGFP evenly distributed throughout the cytoplasm and the nucleoplasm but not the nucleolar, on the contrary AcGFP-N protein localized to both the cytoplasm and nucleolus but not the nucleus in Vero E6 cells (Figure 3). From the above results we hypothesize that there is a NoLS in N, which can guide exogenous protein to the nucleolus. To determine the exact NoLS, the AcGFP-tagged truncated different regions of the N protein were transfected into Vero E6 cells, which did not destroy the integrity of the signals. In our observation, AcGFP-NR1 mainly localized to nucleolar structures, AcGFP-NR2 localized predominantly to the cytoplasm and appeared also to accumulate in the nucleolus to the low level as the cytoplasm, whereas AcGFP-NR3 predominantly localized to the cytoplasm; AcGFP-NR1+2 protein same to AcGFP-N and localized to the cytoplasm and nucleolus, AcGFP-NR2+3 protein localized predominantly in cytoplasmic (Figure 3). This evidence demonstrated that region 2 targeted to the nucleolus with weaker enrichment, while region 1 localized to the nucleolus with stronger enrichment. Thus, we speculated that region 1 have an effect on the nucleolar localization of N. This data also suggested that region 2 and 3 may contain NESs because region 2 and 3 was mainly directed to the cytoplasm. Interestingly, although region 2 and 3 contained predicted pat7 and patx motifs, respectively, they could either have been submissive to the NES or not functional. None of these fusion proteins had a distribution similar to AcGFP only. 

**Figure 4:**
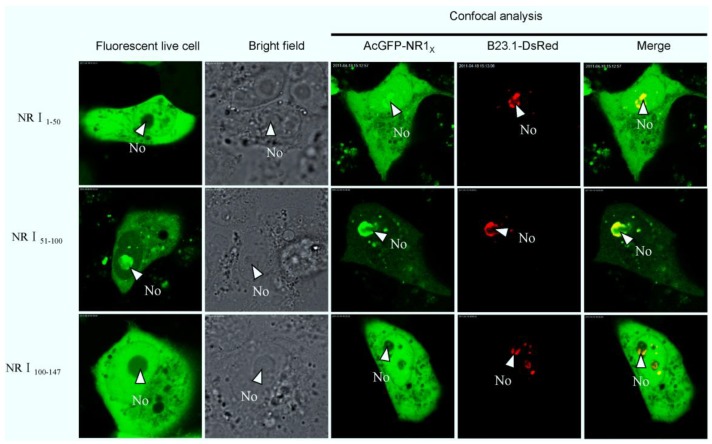
Live cell and confocal microscopy of the sub-cellular localization of fluorescent fusion proteins: AcGFP-NR1_1–50_, AcGFP-N1_51–100_, AcGFP-N1_100–147_. The PEDV fusion peptides are colored green and the B23.1 fusion protein colored red. Merged images are also presented. The nucleolus (No) is arrowed where appropriate.

**Figure 5:**
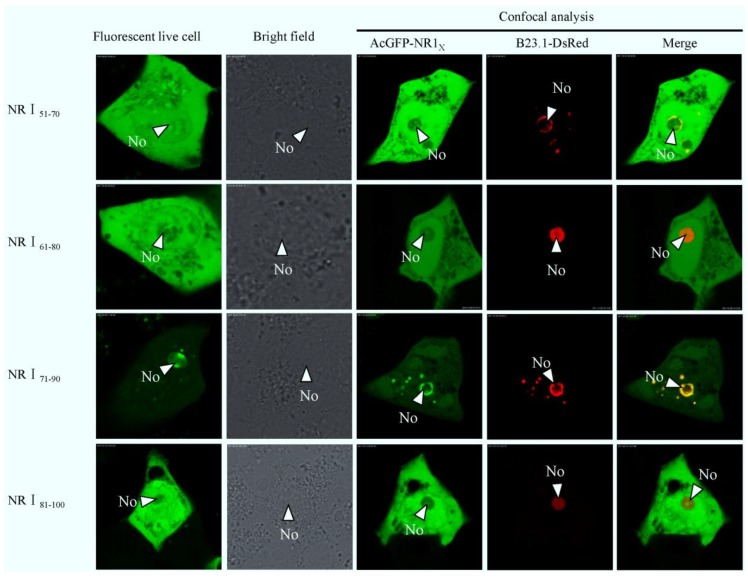
Live cell and confocal microscopy of the sub-cellular localization of fluorescent fusion proteins: AcGFP-NR1_51–70_, AcGFP-NR1_61–80_, AcGFP-NR1_71–90_, AcGFP-NR1_81–100_. The PEDV fusion peptides are colored green and the B23.1 fusion protein colored red. Merged images are also presented. The nucleolus (No) is arrowed where appropriate.

**Figure 6:**
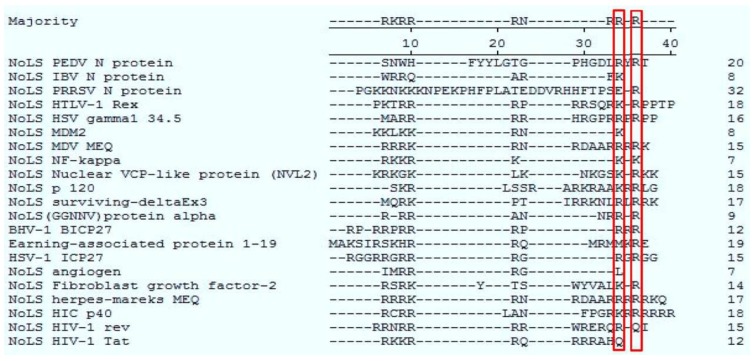
Clustal W analysis of the PEDV N protein NoLS with known cellular and viral NoLSs. Red squares indicate the amino acids of conservation. The cellular and viral NoLSs are described in NoLS IBV N protein [34], NoLS PRRSV N [20], NoLS HTLV-1 Rex [35], NoLS HSV gamma1 34.5 [36], NoLS MDM2 [37], NoLS MDV MEQ [38], NoLS NF-kappa [39], NoLS Nuclear VCP-like protein (NVL2) [40], NoLS p 120 [41], NoLS surviving-deltaEx3 [42], NoLS (GGNNV) protein alpha [43], BHV-1 BICP27 [44], Earning-associated protein 1–19 [45], HSV-1 ICP27 [46], NoLS angiogen [47], NoLS Fibroblast growth factor-2 [48], NoLS herpes-mareks MEQ [38], NoLS HIC p40 [49], NoLS HIV-1 rev [50], NoLS HIV-1 Tat [51].

To further identify the sequence for nucleolar localization in detail, a series of expression constructs containing fragments of region 1 were constructed. Vero E6 cells were cotransfected with, either pAcGFP-NR1_1–50_, pAcGFP-NR1_51–100_, or pAcGFP-NR1_101–147_, and pDsRed-B23.1, analyzed using live cell imaging and confocal microscopy at 24 h post-transfection. The data indicated that AcGFP-NR1_51–100_ colocalized with B23.1, whereas the other two fusion proteins did not (Figure 4). To further refine the amino acids involved in nucleolar localization, 20 amino acid overlapping motifs encompassing amino acids 51–100 were cloned into downstream of AcGFP, creating plasmids pAcGFP-NR1_51–70_, pAcGFP-NR1_61–80_, pAcGFP-NR1_71–90_, and pAcGFP-NR1_81–100_ for the expression of recombinant fusion proteins. Vero E6 cells were cotransfected with these constructs and pDsRed-B23.1, at 24 h post-cotransfection analyzed using live cell imaging and confocal microscopy (Figure 5). The data indicated that AcGFP-NR1_71–90_ localized to the nucleolus and colocalized with B23.1. Therefore, the amino acids at positions 71–90 in PEDV N protein were capable to localize in the nucleolus. Comparison of the PEDV N protein NoLS with known cellular and viral NoLSs showed that the R87 and R89 of the PEDV N protein NoLS might be conserved, although some cellular and viral NoLSs in this site did not contain basic amino acids (Figure 6). Current work is directed at further resolving PEDV NoLS sequence, including the contribution of individual amino acid residues.

### 2.4. Mapping a Functional NES in PEDV N Protein Region 3

**Figure 7:**
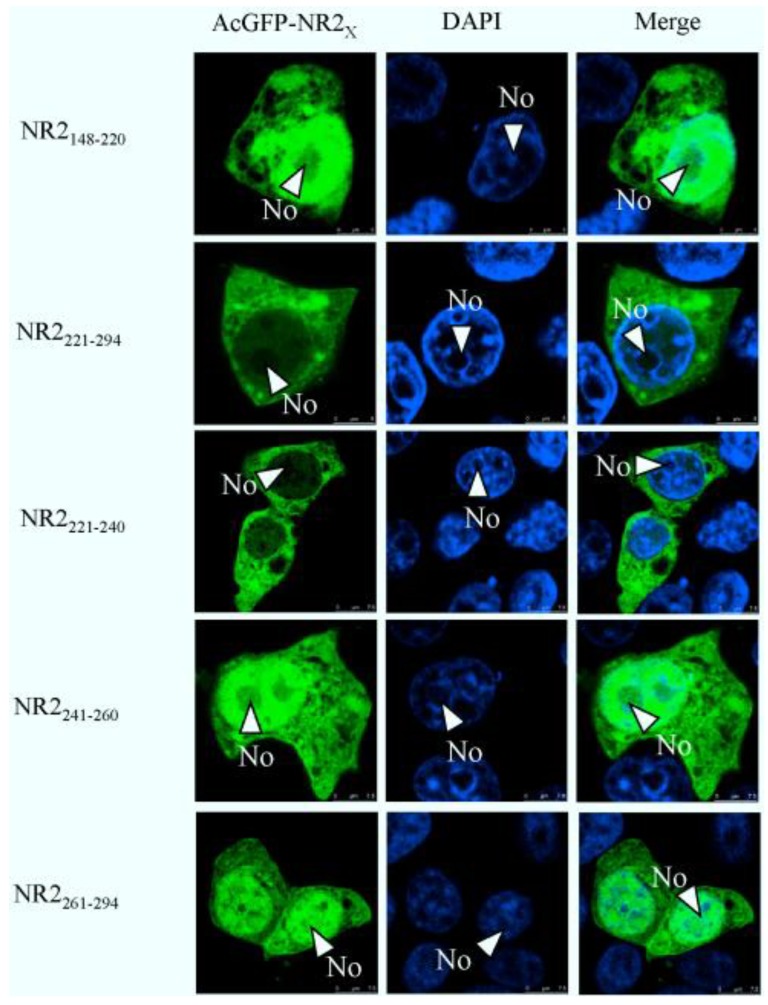
Confocal microscopy of the sub-cellular localization of fluorescent fusion proteins: AcGFP-NR2_148–220_, AcGFP-NR2_221–294_ AcGFP-NR2_221–240_, AcGFP-NR2_241–260_, and AcGFP-NR2_261–294_. Fusion proteins are colored green and nucleus colored blue. Merged images are also presented. The nucleolus (No) is arrowed where appropriate.

**Figure 8:**
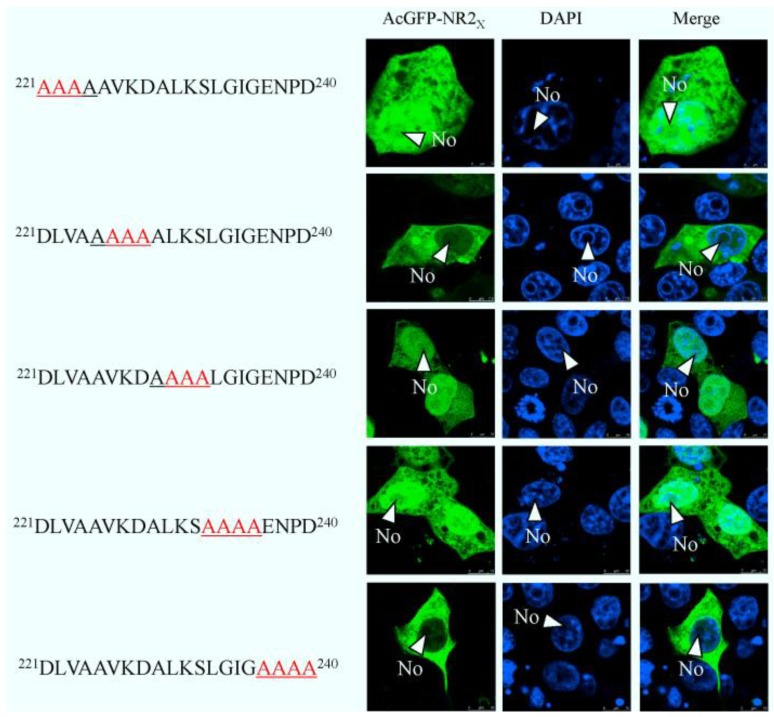
Confocal microscopy of the sub-cellular localization of fluorescent fusion proteins: AcGFP-NR2_221DLVA-AAAA_, AcGFP-NR2_225AVKD-AAA_A, AcGFP-NR2_229ALKS-AAAA_, AcGFP-NR2_233LGIG-AAAA_ and AcGFP-NR2_237ENPD-AAAA_ Fusion proteins are colored green and nucleus colored blue. Merged images are also presented. The nucleolus (No) is arrowed where appropriate.

**Figure 9:**
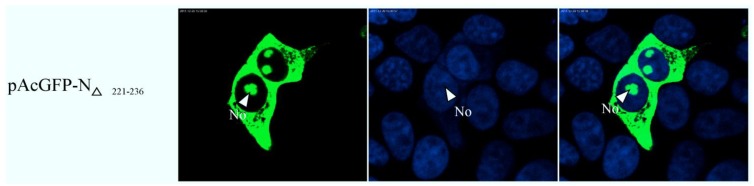
Confocal microscopy of the sub-cellular localization of fluorescent fusion proteins AcGFP-N_∆221–236_. Fusion proteins are colored green and nucleus colored blue. Merged images are also presented. The nucleolus (No) is arrowed where appropriate.

To map the amino acid sequence of region 2 responsible for its nuclear export, similar to the approach used to identify the NoLS in region 1, region 2 was subdivided into two distinct components. Amino acids 148–220 and 221–294 were placed downstream of AcGFP, creating expression vectors pAcGFP-NR2_148–220_ and pAcGFP-NR2_221–294_. These expression plasmids were transfected into Vero E6 cells, the nuclear was stained with DAPI at 24 h post-transfection indicated that amino acids 148–220 directed AcGFP to the cytoplasm and nucleus and had a subcellular localization similar to AcGFP. In contrast, amino acids 221–294 directed AcGFP to the cytoplasm; further investigation revealed that amino acids 241–260 and 261–294, when fused to AcGFP, directed this protein to the cytoplasm and nucleus, whereas amino acids 221–240 directed AcGFP to the cytoplasm (Figure 7). To further define the amino acids involved in cytoplasm trafficking, we conducted a tetra-alanine substitution mutagenesis of amino acids 221–240. These were placed down stream of AcGFP, creating expression plasmids, pAcGFP-NR2_221–224DLVA-AAAA_, pAcGFP-NR2_225–228AVKD-AAAA_, pAcGFP-NR2_229–232ALKS-AAAA_, pAcGFP-NR2_233-236LGIG-AAAA_ and pAcGFP-NR2_237-240ENPD-AAAA_. Therefore, in some cases, the wild-type alanine was not substituted. The data indicated that substituting 221-224DLVA-AAAA, 229-232ALKS-AAAA, and 233-236LGIG-AAAA abolished cytoplasm trafficking. The remaining tetra-alanine substitutions had no effect on cytoplasm trafficking (Figure 8), indicating that amino acids 221 DLVAAVKDALKSLGIG 236 were involved in cytoplasm trafficking. To test whether this amino acid sequence was involved in directing the cytoplasm trafficking of N protein, this motif was deleted in the context of full-length N protein tagged to AcGFP (plasmid pAcGFP-N_∆221–236_). This plasmid was transfected into Vero E6 cells and the subcellular localization of the resulting fusion protein AcGFP-N_∆221–236_ investigated using confocal microscopy. There was no difference at 24 h post-transfection (Figure 9) compared with cells expressing AcGFP-N protein. This data also indicated that the NES identified in region 2 was not necessary for cytoplasm trafficking in PEDV N protein. 

**Figure 10:**
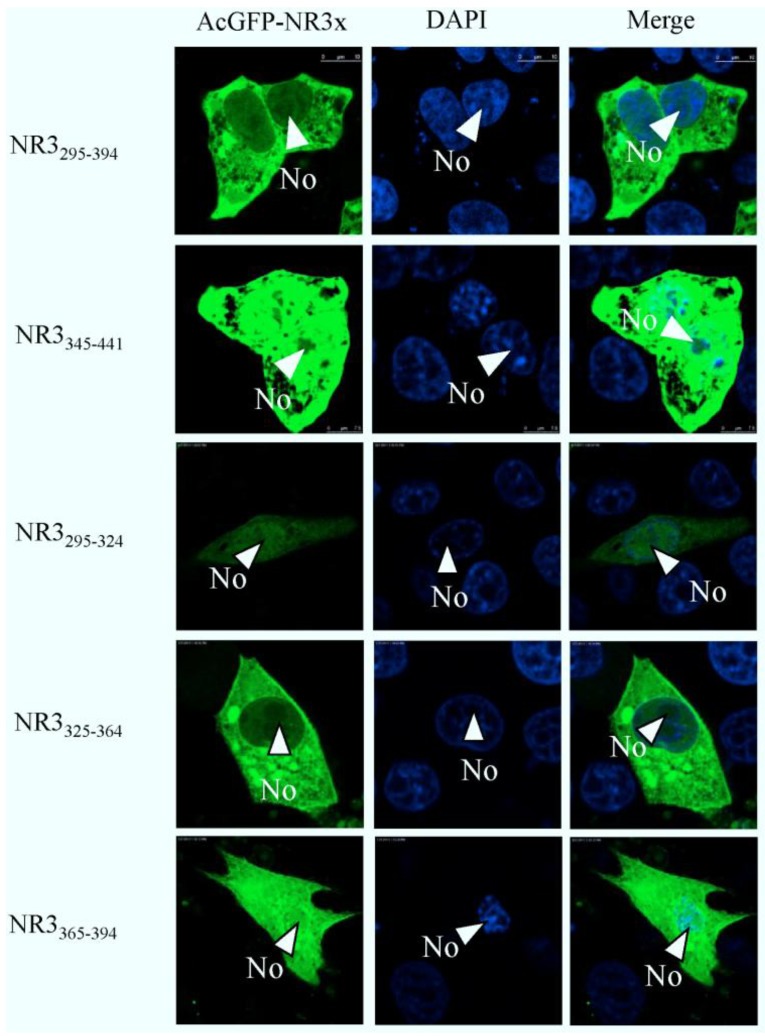
Confocal microscopy of the sub-cellular localization of fluorescent fusion proteins: AcGFP-N_295–394_ and AcGFP-N_345–441_ AcGFP-N_295–324_, AcGFP-N_325–364_, AcGFP-N_365–394_. Fusion proteins are colored green and nucleus colored blue. Merged images are also presented. The nucleolus (No) is arrowed where appropriate.

As region 3 of PEDV N protein localized to the cytoplasm, thus, the similar approach was used to identify the NES in region 3. The data indicated that amino acids 295–394 directed AcGFP to the cytoplasm; further investigation revealed that amino acids 325–364 directed AcGFP to the cytoplasm (Figure 10). Taken together, we proposed that amino acids 325–364 are necessary and sufficient to direct N protein to the cytoplasm, and no other signals are involved.

### 2.5. The Nuclear Export of N Protein Is CRM1 Dependent

**Figure 11:**
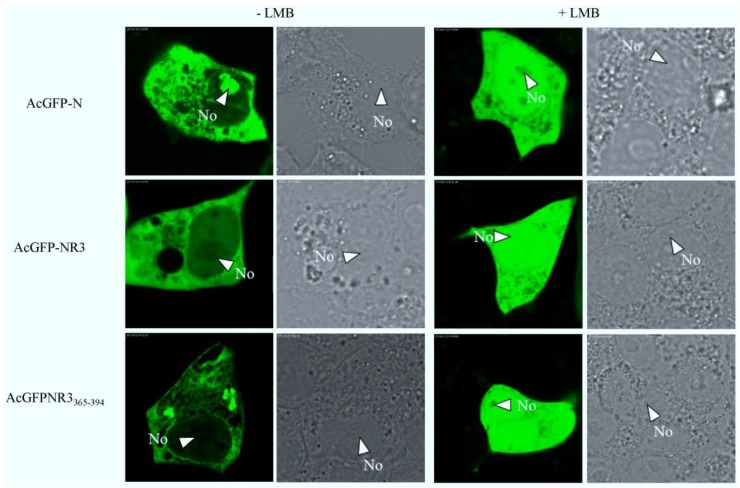
The nuclear export mechanism of PEDV N. Vero E6 cells were transiently transfected with plasmids encoding pAcGFP-N, pAcGFP-NR3, pAcGFPNR3_365-394_, with or without treatment with LMB, and examined live 24 h after transfection by confocal microscopy. Each image is representative of the majority of the cells observed in the same cells. The nucleolus (No) is arrowed where appropriate.

To determine if the PEDV N protein was exported via the CRM1-mediated pathway, the subcellular localization of the pAcGFP-N, pAcGFP-NR3 or pAcGFP-NR3_325-364_, was compared between Vero E6 cells left untreated or treated with 2.5 ng/mL LMB. As shown in Figure 11, the nucleocytoplasmic shuttling of PEDV N, NR3 and NR3_325–364_ was completely inhibited by LMB. The finding indicates that PEDV N shuttling activity was affected by LMB and, thus, suggests that PEDV N protein was transported via the classical CRM1-dependent pathway.

PEDV is an important pathogen causing viral diarrhea in the swine industry. Although much research has been carried out on the general characteristics of PEDV, few reports have been reported on the functions of the PEDV structural proteins, especially N protein. N protein plays an important role in virus replication and modulation of host cellular machinery, which should be a result of its self-interaction and interaction with other viral and cellular proteins and with virus and host cell nucleic acids. Therefore, it is important to understand the subcellular localization properties of the PEDV N protein.

To enter and export from the nucleus, all molecules or cargoes must traverse a large macromolecular structure called the nuclear pore complex (NPC), which is located in the nuclear envelope. Small molecules up to 40–60 kDa or less than 10 nm in diameter can passively diffuse through the NPC, but if proteins are larger than this size-exclusion limit and/or are required to move against a concentration gradient, then transport requires energy-driven mechanisms [52]. In this case, most proteins should contain the appropriate trafficking motifs, such as NLS. The rules and signals that govern the nuclear localization of proteins are well defined. NLSs can be classified into several categories, including the pat4 and pat7 motifs and bipartite NLSs, are composed of basic amino acids of a given sequence length. The pat4 motif consists of a continuous stretch of (usually) four basic amino acids, and the pat7 motif starts with a proline residue and is followed by six amino acids [32,53]. By contrast, the signals that govern nucleolar localization and retention are not well defined [26]. The motifs involved are usually rich in arginine and lysine residues; however there is no immediately obvious consensus sequence or structure. Proteins that localize to the nucleolus can also have nuclear-import motifs. The NoLSs that have been identified so far can be grouped into those that contain single motif and those that contain multiple motifs [54].

In this study, the living cells fluorescence microscopy and confocal microscopy were employed for investigating the subcellular localization and nuclear import and export mechanisms of PEDV N protein. It is well known that living cells fluorescence microscopy and confocal microscopy has an advantage over conventional *in vitro* nuclear transport assays in that cells are not physically damaged by microinjection, detergent, or mechanical perforation, this means that cellular components important for trafficking, such as nuclear import and export receptors, the MT network, intact [55,56]. Our results indicated that PEDV N protein mainly distributes throughout the cytoplasm with localization to a substructure within the nucleolus in infected cells. Similar results were also obtained in transfected cells. 

To eliminate the influence of charged protein migration to the nucleolus post-fixation, and to investigate the nuclear (or nucleolar) localization of PEDV N protein and the functions of these motifs in more detail, we generated constructs that express the protein (or parts of the protein) as a fusion with enhanced green fluorescent protein. The protein could then be detected by direct fluorescence using both live-cell and confocal microscopy. No difference in the localization of either protein was observed between different cell lines, and the presence of a fluorescent tag at either the N terminus or C terminus of N protein did not affect the localization of the fusion protein compared with native protein [2,4]. Furthermore, the B23.1 gene was amplified from the Vero E6 cells and then fused with DsRed in order to visualize the nucleolus. 

Transfection assay results indicated that AcGFP localized predominately to the cytoplasm and the nucleus, but not the nucleolus. The characteristics of AcGFP-N protein localized to either the cytoplasm alone or the cytoplasm and nucleolus, with a maximum of 60% of transfected cells exhibiting this phenotype at 24 h. Our studies indicated that fusing AcGFP with PEDV N protein increased the molecular weight of this protein (82 kDa) above the size exclusion limit of the nuclear pore complex, and it could not diffuse passively through the NPC. This result suggested that there must be some signals in N protein that determined the nucleolar localization. A number of viruses and viral proteins can disrupt nucleolar architecture [57], and the N protein of coronavirus can also localize to the nucleolus in a cell cycle dependent manner and this may be related to dynamic trafficking [58]. A previous study of SARS-CoV indicated the N protein inhibited B23 phosphorylation and might influence ribosome biogenesis to suppress host gene expression and create a more favorable milieu for virus survival [25], so the N protein of PEDV might take part in some cellular process. 

To investigate whether PEDV N protein contained a NoLS, the protein was expressed as a series of single and overlapping regions. This preliminary analysis indicated that PEDV N protein contained a NoLS in region 1. Deletion mutagenesis delineated amino acid PEDV N 71–90, a 20 aa motif that modulated nucleolar localization. Furthermore, comparison with cellular and viral NoLSs, the data indicated that R87 and R89 may be critical for the nucleolar localization. For the site of replication and virus assembly is the cytoplasm, if the subcellular localization of the nucleocapsid protein is in the nucleolar, then the protein is not available for RNA synthesis, encapsidation and assembly, and therefore progeny virus production might be less efficient. However, if such proteins do target the nucleolar as part of a virus replication strategy then they will contain appropriate targeting signals. Thus, these will include not only NLSs and NoLSs, but perhaps more importantly NES. By using a series of N deletion mutants fused to the AcGFP and tetra-alanine substitution mutagenesis, we identified two NES site in region 2 (aa221–236) and region 3 (aa325–364), but NES in region 2 was deleted in the context of full length N protein did not affect cytoplasm trafficking, suggesting that the NES presented in region 2 submissive to the NES in region 3 or not function. Furthermore, PEDV N protein nucleocytoplasmic shuttling was specifically blocked by LMB treatment suggested that PEDV N protein is transported via the classical CRM1-dependent pathway.

## 3. Experimental Section

### 3.1. Cells Culture and Virus Infection

Vero E6 was grown and maintained in Dulbecco’s modified Eagle’s medium supplemented with 10% heat-inactivated fetal calf serum and penicillin-streptomycin, and incubated at 37 °C in 5% CO_2_. Vero E6 cells were infected with PEDV strain CV777 kindly provided by Pensaert M B at MOI = 1, and were seeded and after 24 h for both indirect immunofluorescence assay and Western blot analysis.

### 3.2. Construction of Plasmids

All enzymes used for cloning procedures were purchased from Takara (Dalian, China) except T4 DNA ligase from New England Biolabs (Ipswich, MA, USA). pDsRed-B23.1 encodes a fusion of DsRed protein to the amino terminus of B23.1 and pAcGFP-N encodes a *C*-terminally GFP-epitope-tagged version of PEDV N and has been previously described [59]. The regions and subregions were amplified by PCR using pAcGFP-N as a template. Primers incorporated 5' *Bam*H I site and 3' *Xho*1 used for cloning into pET30a; Primers incorporated 5' *Kpn* I site and 3' *Xho* I site used for cloning into pcDNA3.1 and the other primers incorporated 5' *Xho*l I site and 3' *Kpn* I site used for cloning into pAcGFP-C1. The cloning procedures for constructing most of recombinant plasmids were similar and all primers are listed in Table 1. At all times, numbers used in primer or construct names denoting amino acid numbers refer to their position on the full length N protein. For the Tetra-alanine substitution, each pair of oligonucleotide strands was annealed, and the resultant double-stranded DNA fragments were cloned into the vector pAcGFP-C1. Additionally, the NES of NR2 was deleted in the context of full-length N protein by overlapping PCR using forward primer 1-U and reverse primer 1-L (Table 1) to generate one PCR product and forward primer 2-U and reverse primer 2-L (Table 1) to generate the second PCR product. A second round of PCR was performed using both PCR products as templates and 1-U and 2-L as the forward and reverse primer, respectively. The resulting product was subcloned into pAcGFP-C1. All plasmid were confirmed by sequencing analysis. 

**Table 1:** Primers for constructing recombinant plasmids.

Name of plasmids	Forward primer(5'→3')	Reverse primer(5'→3')
pET30a-N	CAGAGGATCCATGGCTTCTGTCAGC	CAAACTCGAGTTAATTTCCTGTATC
pcDNA3.1-N	AGAGGTACCATGGCTTCTGTCAGCTTTCAG	GCGCTCGAGTTAATTTCCTGTATCGAAGAT
pAcGFP-NR1+2	GCACTCGAGCTATGGCTTCTGTCAGCTTTC	GCCCCATCTGGTACCTTAGCAAGCTGCTAC
pAcGFP-NR2+3	TTCACCTCGAGCTATGCGTAGCAGGAGT	GCCGGTACCTTAATTTCCTGTATCGAAGAT
pAcGFP-NR1	GCACTCGAGCTATGGCTTCTGTCAGCTTTC	GCCACGACTGGTACCTTACGAATTTGCACG
pAcGFP-NR2	TTCACCTCGAGCTATGCGTAGCAGGAGT	GCCCCATCTGGTACCTTAGCAAGCTGCTAC
pAcGFP-NR3	TAGCGCTCGAGCTATGTTCGGACCCAGAGG	GCCGGTACCTTAATTTCCTGTATCGAAGAT
pAcGFP-NR1_1–50_	GCACTCGAGCTATGGCTTCTGTCAGCTTTC	ATTGGTACCTTAAATTTGCTGGTCCTTATT
pAcGFP-NR1_51–100_	ACCCTCGAGTTGGGTACTGGAATGAGCAAA	CTTGGTACCTTATTT AGCAACCCAGAAAAC
pAcGFP-NR1_101–147_	GGGCTCGAGAAGAAGGCGCAAAGACTGAAC	GCCACGACTGGTACCTTACGAATTTGCACG
pAcGFP-NR1_51–70_	ACCCTCGAGTTGGGTACTGGAATGAGCAAA	ATGGGTACCTTAAGGTTGTTCAATTCGCTC
pAcGFP-NR1_61–80_	TTCCTCGAGTTATGCGCCGTGGTGAGCGAA	CGCGGTACCTTATGTTCCGAGGTAGTAGAAATG
pAcGFP-NR1_71–90_	TTGCTCGAGTTTCCAATTGGCATTTCTACT	GCCGGTACCTTAAGTCCTATAACGGAGGTCGCCGTG
pAcGFP-NR1_81–100_	ACCCTCGAGTTGGACCTCACGGCGACCTCC	CTTGGTACCTTATTT AGCAACCCAGAAAAC
pAcGFP-NR2_148–220_	GCACTCGAGCTCGTAGCAGGAGTCGTGGCAA	GCCGGTACCTTAATCGCGTGATG
pAcGFP-NR2_221–294_	GCACTCGAGCTGATCTGGTGGCTGCTGTCAA	GCCGGTACCTTAGCAAGCTGCTACGCTATTTTC
pAcGFP-NR2_221–240_	GCACTCGAGCTGATCTGGTGGCTGCTGTCAA	GCCGGTACCTTAGTCAGGATTTTCTCCA
pAcGFP-NR2_241–260_	GCACTCGAGCTAGGCATAAGCAACAGCAGAA	GCCGGTACCTTATGTATTTTTGCCGCTGTTGTC
pAcGFP-NR2_261–294_	GCACTCGAGCTCCTAAGAAGAACAAATCCA	GCCGGTACCTTAGCAAGCTGCTACGCTATTTTC
pAcGFP-NR3_295–394_	GCACTCGAGCTTTCGGACCCAGAGGGGGCTT	GCCGGTACCTTACGTGGTTTCACGCTTGTTCT
pAcGFP-NR3_345–441_	GCACTCGAGCTGACTCTTACGAGATTAC	GCCGGTACCTTAATTTCCTGTATCGAAGAT
pAcGFP-NR3_295–324_	GCACTCGAGCTTTCGGACCCAGAGGGGGCTT	GCGGTACCACTGGCGATCT
pAcGFP-NR3_325–364_	GCACTCGAGCTTTAGCACCAAAT	GCCGGTACCAACATTTGGATCTGACT
pAcGFP-NR3_365–394_	GCACTCGAGCTGAGCTTCTTGTTTCACAGG	GCCGGTACCCGTGGTTTCACGCTTGTTCT
pAcGFP-NR2_221–224DLVA-AAAA_	TCGAGCTGCTGCTGCTGCTGCTGTCAAGGATGCACTTAAATCTTTGGGTATTGGAGAAAATCCTGACTAAGGTAC	CTTAGTCAGGATTTTCTCCAATACCCAAAGATTTAAGTGCATCCTTGACAGCAGCAGCAGCAGCAGC
pAcGFP-NR2_225–228AVKD-AAAA_	TCGAGCTGATCTGGTGGCTGCTGCTGCTGCTGCACTTAAATCTTTGGGTATTGGAGAAAATCCTGACTAAGGTAC	CTTAGTCAGGATTTTCTCCAATACCCAAAGATTTAAGTGCAGCAGCAGAAGCAGCCACCAGATCAGC
pAcGFP-NR2_229–232ALKS-AAAA_	TCGAGCTGATCTGGTGGCTGCTGTCAAGGATGCTGCTGCTGCTTTGGGTATTGGAGAAAATCCTGACTAAGGTAC	CTTAGTCAGGATTTTCTCCAATACCCAAAGCAGCAGCAGCATCCTTGACAGCAGCCACCAGATCAGC
pAcGFP-NR2_233–236LGIG-AAAA_	TCGAGCTGATCTGGTGGCTGCTGTCAAGGATGCACTTAAATCTGCTGCTGCTGCTGAAAATCCTGACTAAGGTAC	CTTAGTCAGGATTTTCAGCAGCAGCAGCAGATTTAAGTGCATCCTTGACAGCAGCCACCAGATCAGC
pAcGFP-NR2_237–240ENPD-AAAA_	TCGAGCTGATCTGGTGGCTGCTGTCAAGGATGCACTTAAATCTTTGGGTATTGGAGCTGCTGCTGCTTAAGGTAC	CTTAAGCAGCAGCAGCTCCAATACCCAAAGATTTAAGTGCATCCTTGACAGCAGCCACCAGATCAGC
pAcGFP-N _∆221–236_	GCCTCGAGATGGCTTCTGTCAGCTTT(1-U)	ATGCCTGTCAGGATTTTCATCGCGTGATGTCATT(1-L)
	GGAATGACATCACGCGATGAAAATCCTGACAGGCATAA(2-U)	GCGGTACCTTAATTTCCTGTGTC(2-L)

### 3.3. Generation of Polyclonal Antisera in Mouse

The recombinant 6× His-tagged N protein was expressed in *E. coli* BL21 (DE3) cells after induction with 1 mM IPTG for 5 h in LB-medium at 37 °C. The recombinant protein was purified by a GravitrapTM affinity column (GE Healthcare, Bio-Sciences, Piscataway, NJ, USA) according to the manufacturer’s instructions. 

BALB/c mice were immunized with 0.1~0.2 mg of purified recombinant N protein injected subcutaneously at multiple sites on the back. Booster injections were given two and four weeks later. Blood was drown from the mouse at the fifth week following the immunization, and the blood was allowed to clot at 4 °C and the antiserum was recovered by centrifugation at 5,000× *g* for 10 min at 4 °C. A control serum was made by injecting normal saline under the same conditions. The animal experiment was approved by Harbin Veterinary Research Institute and performed in accordance with animal ethics guidelines and approved protocols. The animal Ethics Committee approval number is Heilongjiang-SYXK-2006-032.

### 3.4. Transfection and Western Blotting

On the day before transfection, 0.5~2 × 10^5^ cells grown on 35 mm culture dishes so that 60%–80% confluent cell monolayers were transfected with plasmid DNA with Lipofectamine™ 2000 (Invitrogen, Groningen, Netherlands) according to the instructions of the manufacturer. Protein samples were collected 48 h after transfection by direct lysis of the cells in 5× SDS protein sample buffer with 5% 2-mercaptoethanol. The samples were boiled for 5 min, resolved with 12% SDS-PAGE, and blotted with the following antibodies. For the infection and transfection protein samples: the N protein polyclonal antiserum was the first antibody and IRDyeTM700DX Conjugated Affinity Purified anti-Mouse IgG (H&L)(Mouse) (LI-COR Biosciences, Lincoln, NE, USA) was the second antibody; The images were acquired by The ODYSSEYTM Infrared Imaging System (LI-COR).

### 3.5. Indirect Immunofluorescence Assay

Vero E6 Cells were grown on coverslips and fixed 24 h post-infection (or post-transfection) with 50% methanol-50% acetone for analysis by indirect immunofluorescence using mouse anti-PEDV N polyclonal sera (1:50 dilution) followed by fluorescein isothiocyanate (FITC)-labeled goat anti-mouse antibody (Sigma, St Louis, MO, USA). The cells were washed twice with phosphate-buffered saline (PBS) and subjected to perforation using 0.2% Triton X-100. Then the cells were stained with propidium iodide (PI) (50 μg/mL) (Sigma) to visualize nuclear DNA. Samples were analyzed using fluorescence microscopy.

### 3.6. Confocal Microscopic Analysis

For AcGFP and DsRed fusion expression constructs, co-transfection analyses were carried out. At 24 h post-transfection, subcellular localization properties in living cells were analyzed by laser confocal scanning microscope (Leica Laser Technik, Heidelberg, Germany) with appropriate filters. In order to better display the results at the time of identify the NES in the regions of NR2 and NR3, at 24 h after transfection, cells on glass cover slips were rinsed with PBS and subjected to fixation using 50% methanol-50% acetone for 30 min and permeabilized with 0.2% Triton X-100. Then the nuclear was stained with 4',6-diamidino-2-phenylindole (DAPI) (0.05μg /mL) (Sigma) and analyzed by confocal microscope.

### 3.7. LMB Treatment

Cells were transfected with pAcGFP-N, pAcGFP-NR3, or pAcGFP-NR3_325–364_. At 18 h post-transfection, cells were washed with PBS followed by either treated with 2.5 ng/mL LMB diluted with medium or left in unsupplemented cell culture medium for a further 6 h, then analyzed by used confocal microscope.

## 4. Conclusions

A NoLS (aa71–90) and a functional NES (aa325–364) of PEDV N protein were identified for the first time. Additionally, the N protein was demonstrated to transport between the nucleolus and the cytoplasm through the NES by CRM1-dependent pathway.

## References

[1] Egberink H.F., Ederveen J., Callebaut P., Horzinek M.C. (1988). Characterization of the structural proteins of porcine epizootic diarrhea virus, strain CV777. Am. J. Vet. Res..

[2] Hiscox J.A., Wurm T., Wilson L., Britton P., Cavanagh D., Brooks G. (2001). The coronavirus infectious bronchitis virus nucleoprotein localizes to the nucleolus. J. Virol..

[3] Brian D., Baric R.S., Enjuanes L. (2005). Coronavirus genome structure and replication. Coronavirus Replication and Reverse Genetics.

[4] You J., Dove B.K., Enjuanes L., DeDiego M.L., Alvarez E., Howell G., Heinen P., Zambon M., Hiscox J.A. (2005). Subcellular localization of the severe acute respiratory syndrome coronavirus nucleocapsid protein. J. Gen. Virol..

[5] You J.H., Reed M.L., Hiscox J.A. (2007). Trafficking motifs in the SARS coronavirus nucleocapsid protein. Biochem. Biophys. Res. Commun..

[6] Li F.Q., Xiao H., Tam J.P., Liu D.X. (2005). Sumoylation of the nucleocapsid protein of severe acute respiratory syndrome coronavirus. FEBS Lett..

[7] Li Y.H., Li J., Liu X.E., Wang L., Li T., Zhou Y.H., Zhuang H. (2005). Detection of the nucleocapsid protein of severe acute respiratory syndrome coronavirus in serum: Comparison with results of other viral markers. J. Virol. Methods.

[8] Surjit M., Liu B., Chow V.T., Lal S.K. (2006). The nucleocapsid protein of severe acute respiratory syndrome-coronavirus inhibits the activity of cyclincyclin-dependent kinase complex and blocks S phase progression in mammalian cells. J. Biol. Chem..

[9] Spiegel M., Pichlmair A., Martinez-Sobrido L., Cros J., García-Sastre A., Haller O., Weber F. (2005). Inhibition of beta interferon induction by severe acute respiratory syndrome coronavirus suggests a two-step model for activation of interferon regulatory factor-3. J. Virol..

[10] Zheng B., He M.L., Wong K.L., Lum C.T., Poon L.L., Peng Y., Guan Y., Lin M.C., Kung H.F. (2004). Potent inhibition of SARS associated coronavirus (SCoV) infection and replication by type I interferons (IFN-alpha/beta) but not by type II interferon (IFN-gamma). J. Interferon Cytokine Res..

[11] Yan X., Hao Q., Mu Y., Timani K.A., Ye L., Zhu Y., Wu J. (2006). Nucleocapsid protein of SARS-CoV activates the expression of cyclooxygenase-2 by binding directly to regulatory elements for nuclear factor-kappa B and CCAAT/enhancer binding protein. Int. J. Biochem. Cell Biol..

[12] Kopecky-Bromberg S.A., Martinez-Sobrido L., Frieman M., Baric R.A., Palese P. (2007). Severe acute respiratory syndrome coronavirus open reading frame (ORF) 3b, ORF 6, and nucleocapsid proteins function as interferon antagonists. J. Virol..

[13] He R., Leeson A., Andonov A., Li Y., Bastien N., Cao J., Osiowy C., Dobie F., Cutts T., Ballantine M. (2003). Activation of AP-1 signal transduction pathway by SARS coronavirus nucleocapsid protein. Biochem. Biophys. Res. Commun..

[14] Surjit M., Liu B., Kumar P., Chow V.T., Lal S.K. (2004). The nucleocapsid protein of the SARS coronavirus is capable of self-association through a *C*-terminal 209 amino acid interaction domain. Biochem. Biophys. Res. Commun..

[15] Surjit M., Liu B., Jameel S., Chow V.T., Lal S.K. (2004). The SARS coronavirus nucleocapsid protein induces actin reorganization and apoptosis in COS-1 cells in the absence of growth factors. Biochem. J..

[16] Zhang L., Wei L., Jiang D., Wang J., Cong X., Fei R. (2007). SARS-CoV nucleocapsid protein induced apoptosis of COS-1 mediated by the mitochondrial pathway. Artif. Cells Blood Substit. Immobil. Biotechnol..

[17] Luo C., Luo H., Zheng S., Gui C., Yue L., Yu C., Sun T., He P., Chen J., Shen J. (2004). Nucleocapsid protein of SARS coronavirus tightly binds to human cyclophilin A. Biochem. Biophys. Res. Commun..

[18] Sonia Z., Isabel S., Jose L.M., Patricia S., Juan P.D., Luis E. (2007). Coronavirus nucleocapsid protein is an RNA chaperone. Virology.

[19] Lam Y.W., Trinkle M.L., Lamond A.I. (2005). The nucleolus. J. Cell Sci..

[20] Rowland R.R., Yoo D. (2003). Nucleolar-cytoplasmic shuttling of PRRSV nucleocapsid protein: A simple case of molecular mimicry or the complex regulation by nuclear import, nucleolar localization and nuclear export signal sequences. Virus Res..

[21] Wurm T., Chen H., Hodgson T., Britton P., Brooks G., Hiscox J.A. (2001). Localization to the nucleolus is a common feature of coronavirus nucleoproteins, and the protein may disrupt host cell division. J. Virol..

[22] Timani K.A., Liao Q., Ye L., Zeng Y., Liu J., Zheng Y., Ye L., Yang X., Lingbao K., Gao J. (2005). Nuclear/nucleolar localization properties of *C*-terminal nucleocapsid protein of SARS coronavirus. Virus Res..

[23] Chen H., Wurm T., Britton P., Brooks G., Hiscox J.A. (2002). Interaction of the coronavirus nucleoprotein with nucleolar antigens and the host cell. J. Virol..

[24] Yoo D., Wootton S.K., Li G., Song C., Rowland R.R. (2003). Colocalization and interaction of the porcine arterivirus nucleocapsid protein with the small nucleolar RNA-associated protein fibrillarin. J. Virol..

[25] Zeng Y., Ye L., Zhu S., Zheng H., Zhao P., Cai W., Su L., She Y., Wu Z. (2008). The nucleocapsid protein of SARS-associated coronavirus inhibits B23 phosphorylation. Biochem. Biophys. Res. Commun..

[26] Carmo-Fonseca M., Mendes-Soares L., Campos I. (2000). To be or not to be in the nucleolus. Nat. Cell Biol..

[27] Bednenko J., Cingolani G., Gerace L. (2003). Nucleocytoplasmic transport: Navigating the channel. Traffic..

[28] Gorlich D., Mattaj I.W. (1996). Nucleocytoplasmic transport. Science.

[29] La Cour T., Gupta R., Rapacki K., Skriver K., Poulsen F.M., Brunak S. (2003). NESbase version 1.0: A database of nuclear export signals. Nucleic Acids Res..

[30] Matsuyama A., Arai R., Yashiroda Y., Shirai A., Kamata A., Sekido S., Kobayashi Y., Hashimoto A., Hamamoto M., Hiraoka Y. (2006). ORFeome cloning and global analysis of protein localization in the fission yeast *Schizosaccharomyces pombe*. Nat. Biotechnol..

[31] Fornerod M., Ohon M., Yoshida M., Mattaj I.W. (1997). CRM1 is an export receptor for leucine-rich nuclear export signals. Cell.

[32] Cokol M., Nair R., Rost B. (2000). Finding nuclear localization signals. EMBO Rep..

[33] Nakai K., Horton P. (1999). PSORT: A programme for detecting sorting signals in proteins and predicting their subcellular localization. Trends Biochem. Sci..

[34] Reed M.L., Dove B.K., Jackson R.M., Collins R., Brooks G., Hiscox J.A. (2006). Delineation and modelling of a nucleolar retention signal in the coronavirus nucleocapsid protein. Traffic.

[35] Siomi H., Shida H., Nam S.H., Nosaka T., Maki M., Hatanaka M. (1988). Sequence requirements for nucleolar localization of human T cell leukemia virus type I pX protein, which regulates viral RNA processing. Cell.

[36] Cheng G., Brett M.E., He B. (2002). Signals that dictate nuclear, nucleolar, and cytoplasmic shuttling of the gamma (1) 34.5 protein of herpes simplex virus type 1. J. Virol..

[37] Lohrum M.A., Ashcroft M., Kubbutat M.H., Vousden K.H. (2000). Identification of a cryptic nucleolar-localization signal in MDM2. Nat. Cell Biol..

[38] Liu J.L., Lee L.F., Ye Y., Qian Z., Kung H.J. (1997). Nucleolar and nuclear localization properties of a herpesvirus bZIP oncoprotein, MEQ. J. Virol..

[39] Birbach A., Bailey S.T., Ghosh S., Schmid J.A. (2004). Cytosolic, nuclear and nucleolar localization signals determine subcellular distribution and activity of the NF-kappa B inducing kinase NIK. J. Cell Sci..

[40] Nagahama M., Hara Y., Seki A., Yamazoe T., Kawate Y., Shinohara T., Hatsuzawa K., Tani K., Tagaya M. (2004). NVL2 is a nucleolar AAA-ATPase that interacts with ribosomal protein L5 through its nucleolar localization sequence. Mol. Biol. Cell.

[41] Guo H., Ding Q., Lin F., Pan W., Lin J., Zheng A.C. (2009). Characterization of the nuclear and nucleolar localization signals of bovine herpesvirus-1 infected cell protein 27. Virus Res..

[42] Valdez B.C., Perlaky L., Henning D., Saijo Y., Chan P.K., Busch H. (1994). Identification of the nuclear and nucleolar localization signals of the protein p120. J. Biol. Chem..

[43] Song Z., Wu M. (2005). Identification of a novel nucleolar localization signal and a degradation signal in Survivin-deltaEx3: A potential link between nucleolus and protein degradation. Oncogene.

[44] Guo H.S., Ding S.W. (2002). A viral protein inhibits the long range signaling activity of the gene silencing signal. EMBO J..

[45] Kim H., Chang D.J., Lee J.A., Lee Y.S., Kaang B.K. (2003). Identification of nuclear/nucleolar localization signal in Aplysia learning associated protein of slug with a molecular mass of 18 kDa homologous protein. Neurosci. Lett..

[46] Mears W.E., Lam V., Rice S.A. (1995). Identification of nuclear and nucleolar localization signals in the herpes simplex virus regulatory protein ICP27. J. Virol..

[47] Lixin R., Efthymiadis A., Henderson B., Jans D.A. (2001). Novel properties of the nucleolar targeting signal of human angiogenin. Biochem. Biophys. Res. Commun..

[48] Sheng Z., Lewis J.A., Chirico W.J. (2004). Nuclear and nucleolar localization of 18-kDa fibroblast growth factor-2 is controlled by *C*-terminal signals. J. Biol. Chem..

[49] Thebault S., Basbous J., Gay B., Devaux C., Mesnard J.M. (2000). Sequence requirement for the nucleolar localization of human I-mfa domain-containing protein (HIC p40). Eur. J. Cell Biol..

[50] Cochrane A.W., Perkins A., Rosen C.A. (1990). Identification of sequences important in the nucleolar localization of human immunodeficiency virus Rev: Relevance of nucleolar localization to function. J. Virol..

[51] Siomi H., Shida H., Maki M., Hatanaka M. (1990). Effects of a highly basic region of human immunodeficiency virus Tat protein on nucleolar localization. J. Virol..

[52] Lange A., Mills R.E., Lange C.J., Stewart M., Devine S.E., Corbett A.H. (2007). Classical nuclear localization signals: Definition, function, and interaction with importin alpha. J. Biol. Chem..

[53] Macara I.G. (2001). Transport into and out of the nucleus. Microbiol. Mol. Biol. Rev..

[54] Kubota N., Terauchi Y., Miki H., Tamemoto H., Yamauchi T., Komeda K., Satoh S., Nakano R., Ishii C., Sugiyama T. (1999). PPAR γ mediates high-fat diet-induced adipocyte hypertrophy and insulin resistance. Mol. Cell.

[55] Dohner K., Wolfstein A., Prank U., Echeverri C., Dujardin D., Vallee R., Sodeik B. (2002). Function of dynein and dynactin in herpessimplex virus capsid transport. Mol. Biol. Cell.

[56] Giannakakou P., Sackett D.L., Ward Y., Webster K.R., Blagosklonny M.V., Fojo T. (2000). p53 Is associated with cellular microtubules and is transported to the nucleus by dynein. Nat. Cell Biol..

[57] Hiscox J.A. (2007). RNA viruses: Hijacking the dynamic nucleolus. Nat. Rev. Microbiol..

[58] Cawood R., Harrison S.M., Dove B., Reed M.L., Hiscox J.A. (2007). Cell cycle dependent localization of the coronavirus nucleocapsid protein. Cell Cycle.

[59] Lv M.J., Chen J.F., Shi H.Y., Chen C.J., Fan X.P., Shen S.C., Feng L. (2011). Co-localization analysis between porcine epidemic diarrhea virus nucleocapsid protein and nucleolar phosphoprotein B23.1. Acta Microbiol. Sin..

